# Cold regulation of plastid ascorbate peroxidases serves as a priming hub controlling ROS signaling in *Arabidopsis thaliana*

**DOI:** 10.1186/s12870-016-0856-7

**Published:** 2016-07-20

**Authors:** Jörn van Buer, Jelena Cvetkovic, Margarete Baier

**Affiliations:** Dahlem Center of Plant Sciences, Plant Physiology, Freie Universität Berlin, Königin-Luise-Straße 12-16, 14195 Berlin, Germany

**Keywords:** Priming, Antioxidants, Antioxidant system, Chloroplast, Cold, Elevated light intensity, *Arabidopsis thaliana*, Ascorbate peroxidase

## Abstract

**Background:**

Short cold periods comprise a challenge to plant growth and development. Series of cold stresses improve plant performance upon a future cold stress. This effect could be provoked by priming, training or acclimation dependent hardening. Here, we compared the effect of 24 h (short priming stimulus) and of 2 week long cold-pretreatment (long priming stimulus) on the response of *Arabidopsis thaliana* to a single 24 h cold stimulus (triggering) after a 5 day long lag-phase, to test Arabidopsis for cold primability.

**Results:**

Three types of pretreatment dependent responses were observed: (1) The CBF-regulon controlled gene *COR15A* was stronger activated only after long-term cold pretreatment. (2) The non-chloroplast specific stress markers *PAL1* and *CHS* were more induced by cold after long-term and slightly stronger expressed after short-term cold priming. (3) The chloroplast ROS signaling marker genes *ZAT10* and *BAP1* were less activated by the triggering stimulus in primed plants.

The effects on *ZAT10* and *BAP1* were more pronounced in 24 h cold-primed plants than in 14 day long cold-primed ones demonstrating independence of priming from induction and persistence of primary cold acclimation responses. Transcript and protein abundance analysis and studies in specific knock-out lines linked the priming-specific regulation of *ZAT10* and *BAP1* induction to the priming-induced long-term regulation of stromal and thylakoid-bound ascorbate peroxidase (*sAPX* and *tAPX*) expression.

**Conclusion:**

The plastid antioxidant system, especially, plastid ascorbate peroxidase regulation, transmits information on a previous cold stress over time without the requirement of establishing cold-acclimation. We hypothesize that the plastid antioxidant system serves as a priming hub and that priming-dependent regulation of chloroplast-to-nucleus ROS signaling is a strategy to prepare plants under unstable environmental conditions against unpredictable stresses by supporting extra-plastidic stress protection.

## Background

Temperature varies in diurnal and annual patterns. Additionally, irregular temperature changes can impact on plant performance [1]. Cold slows down e.g., enzyme activities, metabolite mobility, energy metabolism and membrane fluidity, changes the structure of biomolecules, and modifies channel conductivities and plant anatomy [2–7]. Primary cold sensing effects, such as changes in Ca^2+^ fluxes [2, 3], mobilization of transcription factors [4] and photosynthetic signals [5, 8], activate signal transduction processes, modify gene expression activities and improve plant performance [9, 10]. The hierarchically organized ICE1 (*INDUCER OF CBF-EXPRESSION 1*) - CBF (*C-REPEAT-BINDING-FACTOR*) - COR (*COLD-REGULATED GENES*) module mediates a wide range of cold acclimation responses [7, 11–14]. The regulon is supported by the release of the NAC transcription factor NTL6 from the plasma membrane [10], by abscisic acid (ABA), cytokinin and salicylic acid (SA) signaling [15–17] as well as ROS-signaling [18, 19]. The subsequently modified processes improve the tolerance of the organisms to cold and other stresses, including biotic stress [9, 10, 20, 21]. Such response to an enduring stressor is defined as acclimation [12].

After removal of the stressor, deacclimation starts. Cold deacclimation is the faster and the more effective, the shorter the cold period lasted [6]. The ICE1-regulated responses, such as *COR*-gene expression and osmolyte accumulation [7], are quickly lost when the temperatures increase [7, 20, 22]. Secondary and more diversely regulated metabolic changes, such as redox- and carbohydrate controlled ascorbate accumulation [23], decline more slowly. Changes in the tissue structure, which take place upon prolonged cold-exposure, are even widely irreversible [6].

Preparation of organisms by an only occasional and discontinuous stress event for better performance on a future stress is defined as priming [24, 25]. Characteristically, the previous stress event is memorized throughout a stress-free phase (lag-phase). While early definitions of priming asked only for a different (usually, an increased) response to a later stress event [26], the more recent definitions include the criterion that the response of the read-out parameter to the first stress event has to be lost before the next stress is applied [25, 27]. Training (sometimes also called “entrainment”) is defined as a modified response after a series of stress applications. In contrast, priming already changes the response to the second stress event [25, 27]. Accumulation of proteins and metabolites, the turn-over of mRNAs and miRNAs, protein marks as well as histone modifications are discussed to store the information about the previous stress event over the stress-free phase [25, 26, 28–31].

Cold training and priming have been indicated in various plant species by higher freezing tolerance after repetitive short cold treatments [32, 33]. As shown for cucumber [33], six days with temperature rhythms of 2 h 12 °C and 22 h 23 °C increased the cold tolerance gradually. From day 3 onwards, it was higher than in continuously cold-treated plants and caused longer lasting cold tolerance [33]. Li et al. [32] linked higher cold tolerance after six nights at low temperatures to better antioxidant protection of the photosynthetic apparatus and showed higher total plastid superoxide dismutase and total plastid ascorbate peroxidase activities. Comparative transcript abundance analysis in 3 week old Arabidopsis plants, which faced only two 4 °C cold pulses separated by 3 day long lag-phase, showed accumulation of transcripts for components of the photosynthetic electron transport chain, chlorophyll biosynthesis, RUBISCO, ascorbate and starch biosynthesis and signal transduction elements, such as Ca^2+^-binding proteins, Ca^2+^-dependent protein kinases, mitogen-activated protein kinase kinases (MAPKK), ERF/AP2-transcription factors and zinc finger proteins, including ZAT10 and ZAT12 [22]. Stronger activation of these cold acclimation responses by the second cold stimulus was postulated to be a strategy to improve freezing tolerance. The work followed the traditional concepts of priming, as proposed in the review by Bruce et al. [26], according to which the later stimulus is placed after the read-out levels of the primary stress responses have started to decrease, but before the responses were entirely lost.

To test *Arabidopsis thaliana* var. Col-0 for priming effects and the relevance of previous cold acclimation, we compared 4 week old Arabidopsis plants after short-term (STC; 24 h 4 °C) and long-term cold (LTC; 14 days 4 °C) pretreatment by triggering them with a 24 h 4 °C pulse after a 5 day long lag-phase. In the range of evaluated Arabidopsis accessions, Col-0 is one with medium strong cold acclimation mechanisms [21, 34, 35]. Within 24 h, more than 70 % of the cold-induced metabolite changes are lost after a 14 day long cold acclimation period [20]. Glucose, fructose, sucrose, raffinose and proline levels, which strongly increase during cold acclimation, are indistinguishable from pre-cold levels after 3 days of deacclimation [20]. 24 h cold pulses are too short to induce e.g., osmolyte synthesis significantly, to change the thylakoid membrane composition, to reactivate carbon fixation and to change the leaf anatomy [6, 20, 36–38].

Analysis of transcript abundances of a selection of stress-regulated genes identified three types of cold priming. One was specific for LTC, one was more pronounced after LTC- than after STC-treatment and one was stronger regulated in plants previously exposed to a 24 h cold-pulse than in long-term cold-treated ones. We postulate that limiting induction of chloroplast-to-nucleus signaling responses and stronger activation of non-chloroplast-specific stress responses primes plants for future stresses, when cold acclimation responses cannot be fully activated.

## Results

**Fig. 1:**
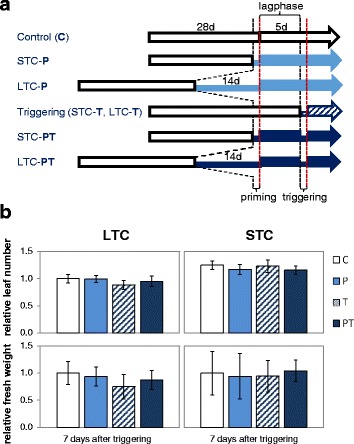
**a** Experimental set-up: 4 week old *Arabidopsis thaliana* plants were cold treated for 24 h (STC) or 14 d (LTC) for priming. After a lag-phase of 5 days, the plants were triggered by applying 24 h cold. The red dotted lines mark the time points “0 h after priming” and “0 h after triggering”. **b** Number of leaves (top) and rosette fresh weight (bottom) in only-primed (P), only-triggered (T) and primed and triggered (PT) Arabidopsis 7 days after triggering relative to the leaf number in untreated control plants (C). left: LTC; right: STC; The bars depict the means ± standard deviation; *n* = 15-20; The significance was tested by ANOVA (Tukey’s test; *p* < 0.05), but no significant differences were observed

To test *Arabidopsis thaliana* for cold priming effects, 28 day old Col-0 plants were cold-treated at 4 °C either for 24 h (short term cold stress; STC) or for 14 days (long term cold stress, LTC). After 5 days at optimal growth temperatures (lag-phase), the primed (“P plants”) and the naïve plants (“C-plants”) were triggered for 24 h at 4 °C (triggered-only plants “T plants” and primed and triggered plants “PT plants”) (Fig. 1a) to test whether the plants memorize the previous cold stress over the 5 day long lag-phase and whether they respond differently to the later cold stimulus after short and long-term cold pretreatment.

### Background parameters

#### Growth parameters

Most Arabidopsis accessions, including Col-0, arrest growth, when they are transferred from optimal growth temperatures to 4 °C [39]. The extent depends on the duration of the cold phase [6]. To control our experimental set-up, we analyzed primed and / or triggered and control plants 7 days after the time-point of triggering for their fresh weights and leaf numbers. The 7 days period was chosen to visualize meristem activities. At this time point, none of our plants had started to bolt. The leaf numbers in cold-treated T and PT plants in the LTC plant set showed a very slight, but not significant (Tukey’s test; *p* < 0.05; *n* = 20) trend towards slower re-activation of leaf formation after triggering (Fig. 1b left). In the STC plant set, all plants developed similarly (Fig. 1b right).

#### Chlorophyll levels

**Fig. 2:**
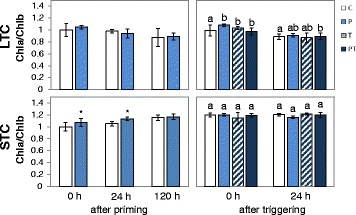
Chlorophyll a / Chlorophyll b ratios (± standard deviation) in LTC- and STC-pre-treated plants after priming and triggering. The crude data are standardized on the values in control plants (C). The data were analyzed by ANOVA (Tukey’s test) and Student’s t-test (*p* < 0.05, *n* = 10–20). Significant differences between primed and unprimed plants are labeled with an asterisk in the left part of the figure. After triggering, significantly different data are labeled with different letters

In the cold and upon re-acclimation to optimal growth temperatures the expression intensity of genes changes, which drive pigment biosynthesis and pigment binding [22]. In the LTC data set, the biological age of primed and unprimed plants differed by 2 weeks (duration of the cold-treatment) (Fig. 1a), which might cause senescence effects and changes in the pigment composition [40, 41]. As an indicator for the thylakoid status, we quantified the chlorophyll levels and analyzed the plants for changes in the chlorophyll-a / chlorophyll-b ratio (chl a/b ratio). The latter provides information, for example, on the relative support of photoreaction centers and antenna proteins [42]. The chl a/b ratio was slightly increased immediately after LTC- and STC-priming (Fig. 2). After short-term priming, it remained elevated for the next day (Fig. 2). Triggering increased the chl a/b ratio very slightly in the LTC primed-plants. However, the comparison of the PT- with the T- values gave no indication for a priming effect.

#### Photosynthetic performance parameters

Chlorophyll-a fluorescence can show differences in photosynthetic regulation processes [43, 44]. The maximum quantum yield of photosystem II (PS-II) (F_V_/F_M_ after dark acclimation; [45]) is an indicator for the excitation potential of photoreaction centers and for damage of PS-II. It was around 0.83 in C, P, T and PT-plants prior to and 1, 2 and 3 days after triggering (data not shown) demonstrating that under the conditions used in this study, the photosystems were not damaged at any time-point of analysis or could be quickly fully regenerated.

**Fig. 3:**
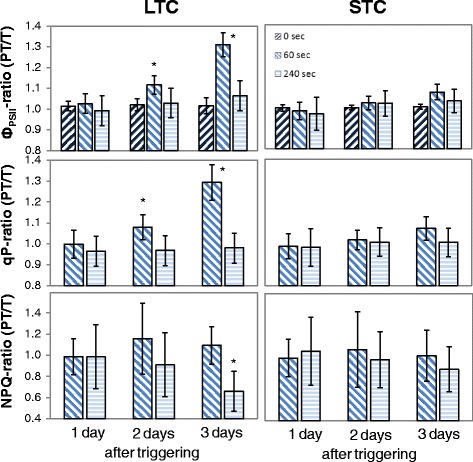
Ratio of quantum yield of photosystem II (F_V_/F_M_ and Φ_PS-II_), photochemical quenching (qP) and non-photochemical quenching (NPQ) in PT-plants vs. T-plants. 1 day, 2 days and 3 days after triggering in LTC- and STC primed and 20 min dark acclimated plants 0 min, 60 s and 240 s after the on-set of actinic light with 3-fold higher light intensity than growth light intensity. The crude data are standardized to the values in control plants (C). The data were analyzed by ANOVA (Tukey’s test; *p* < 0.05, *n* = 5–6). Significant differences to unprimed plants are labeled with an asterisk

To analyze the plants for restrictions in the dynamic of photosynthetic regulation, triggered plants (PT and T) were exposed for 270 s to a 3-fold higher light intensity than the growth light intensity and the effective quantum yield of photosystem II (ϕ_PSII_; [46]) was recorded as an indicator for the short-term light acclimation dynamics. Such moderately elevated light intensity shows differences in metabolic regulation, such as changes in carbohydrate metabolism [47], but avoids strong over-excitation of the photoreaction centers and damage of the photosynthetic electron transport chain. One day after triggering, no difference between primed and non-primed plants was observed (Fig. 3), which excludes persistence of primary priming effects on the photosystem regulation. Two and three days after triggering, higher effective quantum yields of photosystem II (ϕ_PSII_; [46]) and higher photochemical quenching (qP; [48]) were observed between 30 and 120 s after the onset of illumination (Fig. 3 left; 60 s) in the LTC-plants demonstrating that priming supported activation of the electron flux through PS-II by the triggering stimulus. After 240 s, non-photochemical quenching (NPQ; [49]) was decreased. This indicates better energy dissipation activities in LTC-plants [50, 51], such as by stronger activation of C-assimilation in response to the second cold-stimulus. No significant effect (Student’s t-test; *p* < 0.05; *n* = 5–6) was observed in STC-primed plants (Fig. 3 right). Consequently, the short cold-treatments did not change the assimilatory capacities and did not perform super-acclimation after a 5 day long lag-phase, as observed Byun et al. [22] after a 3 day long lag-phase.

### Regulation of stress marker genes

Cold has a wide impact on gene expression in *Arabidopsis thaliana* [52]. To analyze the plants for priming effects, the transcript levels of well-characterized specifically and unspecifically cold regulated genes were compared by qRT-PCR after priming, during the lag-phase and after triggering. The genes *COR15A* (At2g42540; *COLD-REGULATED GENE 15A*), *ZAT10* (At1g27730; *ZINC FINGER OF ARABIDOPSIS THALIANA 10*), *BAP1* (At3g61190; *BON ASSOCIATED PROTEIN* 1), *PAL1* (At2g37040; *PHENYLAMMONIUM LYASE*) and *CHS* (At5g13930; *CHALCONE SYNTHASE*) were pre-selected based on literature on stress regulation of plant genes due to the described specificities for cold and ROS-signaling [53–59]. Furthermore, evidence for pathogen priming was available for *PAL1* [58] and cold-regulated modification of histone methylation of the *COR15A* promoter [60] suggested a potential for priming sensitivity.

The regulation of the five genes was re-tested for the specificity of regulation by comparison of microarray data using the AT_AFFY_AT1-0 data set via the Genevestigator interface [61]. The transcript levels of *COR15A*, *BAP1*, *ZAT10*, *PAL1* and CHS are all induced in response to cold (Genevestigator AT_AFFY_AT1-0 microarray sub-data sets AT-00076 (Knight, unpublished work) and AT-0221 [52]), but were differentially regulated in response to other stresses and signaling pathways: *COR15A* is a direct target of CBF3 [62] and expressed in a CBF2-controlled manner (AT-000176). *BAP1* and *ZAT10* are co-regulated with CBF3-sensitive genes (AT-000220; [63]). *ZAT10* and *CBF3* are regulated by binding of *LOS2* (*LOW EXPRESSION OF OSMOTICALLY RESPONSIVE GENES 2*) to the promoters [14, 64], but are not under CBF2-control (AT-000176).

*BAP1* and *ZAT10* expression is under control of EXECUTER-mediated chloroplast-to-nucleus ROS signaling (AT-000287; [65]), chloroplast ascorbate peroxidase activity (AT-000294; [66]) and ascorbate availability (AT-000469; [67]). The also multifold stress-responsive genes *PAL1* and *CHS* are not influenced by *ICE1*, *CBF2* and *CBF3* (AT-000176; AT-000220) and are inversely regulated to *ZAT10* and *BAP1* in the ascorbate biosynthetic mutant *vtc2* (AT-000469; [67]). Furthermore, *PAL1* and *CHS* are not affected by the EXECUTER-mediated chloroplast-to-nucleus ROS signaling pathway (AT-000294; [66]) and are not sensitive to thylakoid ascorbate peroxidase catalyzed chloroplast antioxidant activity in the *flu1* mutant background (AT-000294; [66]), which promotes generation of chloroplast ROS signals [68].

Based on this information, we used *COR15A* as a cold-specific and chloroplast-independent marker gene, *BAP1* and *ZAT10* as marker genes for chloroplast-to-nucleus ROS signaling and *PAL1* and *CHS* for cold, but not-chloroplast-specific induced stress responses. Throughout the experiment, samples were harvested exactly at the same time of the day, 2 – 2.5 h after the start of the illumination period, to avoid circadian regulation effects and to have strong expression of nuclear genes for chloroplast proteins. For better comparison of responses in the LTC- and STC-plant sets (C-, P-, T- and PT-plants), all transcript data were normalized to the transcript levels in 28 day old naïve plants of each growth series.

#### The cold-response marker COR15A is only priming sensitive to long-term cold

*COR15A* encodes a strongly cold-inducible chloroplast protein [69], which protects the inner envelope of chloroplasts from freezing damage [70]. After the lag-phase, the *COR15A* transcript levels were similar in STC- and LTC-treated plants (P-plants) and untreated control (C) plants demonstrating that the primary cold response of these gene was entirely lost.

**Fig. 4:**
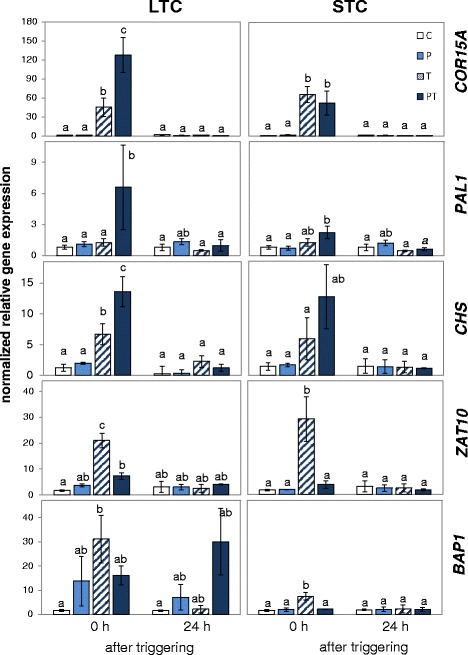
The transcript levels of cold (*COR15A*), pleiotropic stress marker genes (*PAL1* and *CHS*) and chloroplast-ROS marker genes (*ZAT10* and *BAP1*) immediately after 24 h triggering at 4 °C and 24 h later in LTC- and STC-primed plants. The transcript levels were normalized to the respective transcript levels of naïve plants immediately before the on-set of priming. The data were analyzed by ANOVA (Tukey’s test; *p* < 0.05, *n* = 3–6). The small letters differentiate significance groups

Consistent with regulation via the cold-sensitive CBF-regulon [62, 71], the *COR15A* transcript level was strongly increased in response to the 24 h cold stress applied for triggering. LTC-pretreatment (PT-plants) resulted in higher transcript levels than in not pretreated plants (T-plants) showing that the plants memorized the previous cold treatment over a 5 day long lag-phase (Fig. 4). No differences in *COR15A* transcript accumulation were observed between PT- and T-plants in the STC-plant set (Fig. 4). Consequently, only LTC, but not the STC-treatment, caused a priming effect on *COR15A*.

#### PAL1 and CHS are positively priming sensitive

The genes for phenylalanine ammonia lyase 1 (*PAL1*) and chalcone synthase (*CHS*) respond to a wide range of stresses, but are not under control of EXECUTER- and tAPX (thylakoid ascorbate peroxidase)-mediated chloroplast-to-nucleus ROS signaling (AT-000287 [65]; AT-000294 [66]) or the ICE1-CBF-regulon (AT-000220 [63]). Expression is strongly induced upon pathogen infection [72–74], which is mediated by ROS- and redox-shifts in the apoplasm and in the cytosol [75, 76]. The gene products catalyze cold acclimation responses, such as anthocyanin and flavonol biosynthesis [21, 57]. *PAL1* and *CHS* transcript levels were stronger induced in LTC-pretreated plants (PT) than in T-plants immediately after triggering (Fig. 4) demonstrating a positive priming effect similar to the one observed for *PAL1* in response to MPK3- and MPK6-mediated pathogen priming [58].

In the STC plant set, the same trends were observed as in LTC-plants (Fig. 4), but the differences between primed-only and primed and triggered plants (P and PT) were not significant. To reduce background noise coming from the variability of the gene expression intensities prior to triggering, specifically the effects of triggering ((T-C): triggering effect of unprimed plants and (PT-P): triggering effect of primed plants) were compared in a Tukey test for significance (*p* < 0.05). They differed significantly in the STC- and in the LTC-data sets. In both experimental set-ups, the transcript abundance increase caused by priming and by triggering in PT-plants was stronger than the additive value of only priming (P - C) and only triggering (T - C).

#### Marker genes for plastidic ROS-signaling are negatively sensitive to priming

*ZAT10* and *BAP1* were chosen as marker genes for EXECUTER-mediated and chloroplast thylakoid ascorbate peroxidase antagonized chloroplast-to-nucleus ROS-signaling [65, 66]. ZAT10 controls activation of genes involved in extra-plastidic antioxidant protection [56, 77–79] and the adjustment of mesophyll development to environmental conditions [80]. In contrast to the study by Byun et al. [22], in which the two 24 h cold pulses were only separated by 3 days and *ZAT10* transcript levels were stronger induced by the second cold stimulus, *ZAT10* induction by the triggering stimulus was almost fully blocked in PT-plants (compared to the response in not primed T-plants) in our study after a 5 day long lag-phase in the STC-plant set (Fig. 4). In LTC-primed plants, *ZAT10* induction was half-maximal. The result demonstrates that cold priming limits cold induction of *ZAT10* and shows that LTC has a weaker priming effect on *ZAT10* regulation than STC.

Like *ZAT10*, *BAP1* was less induced by the triggering stimulus in STC-primed plants (PT) than in unprimed ones (T) (Fig. 4). The means indicate also slightly lower expression of *BAP1* in PT-plants than in T-plants in the independently grown LTC data set (Fig. 4). The transcript values in the LTC plants did not differ significantly due to higher variability between the biological replicates and partial persistence of BAP1 activation during the lag-phase.

### Ascorbate levels

**Fig. 5:**
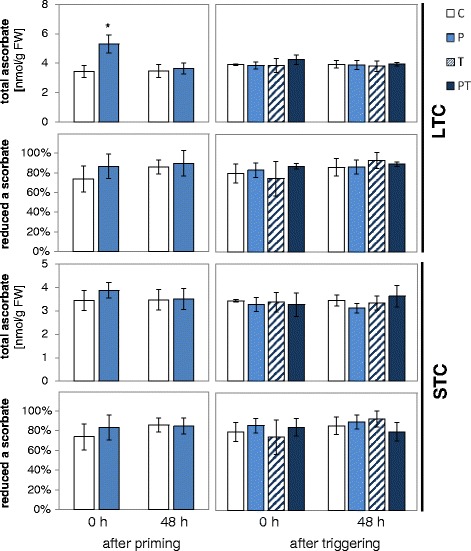
Total ascorbate levels and reduction states of ascorbate in LTC- (*top*) and STC-primed (*bottom*) plants immediately and 2 days after priming and triggering. The data were analyzed by Student’s t-test and by ANOVA (Tukey’s test; *p* < 0.05, *n* = 10). Significant differences to naïve plants are labeled with an asterisk. No significant differences were observed after triggering. For C, P, T and PT plants the same color scheme as in Fig. 1 was applied

Positive priming effects for *PAL1* and *CHS* (Fig. 4), but negative ones for the chloroplast ROS signaling marker genes *ZAT10* and *BAP1* (Fig. 4), indicate a change in the plastid ROS buffering capacity. Comparison of microarray data obtained with Arabidopsis wild-type plants and the ascorbate biosynthetic mutant *vtc2* (AT-000469; [67]) suggests regulation by the availability of ascorbate. As reported by others and ourselves before [23, 81, 82], ascorbate levels increase during long cold periods (Fig. 5 left top). The 24 h long 4 °C treatments used for STC-priming and triggering were not sufficient to change the ascorbate levels (Fig. 5), excluding priming regulation via the ascorbate pool size.

During longer cold periods, the reduction state of the chloroplast stroma increases [83], which affects also the reduction state of the ascorbate pool (ratio of reduced ascorbate and total ascorbate) [23]. In the here presented experiments, the ascorbate pool was only slightly more reduced immediately after LTC-priming, but not after STC-treatment. At all other time points, it was similar irrespective of the treatments and excludes regulation of the ascorbate pool as driving force for the observed priming responses on gene expression.

### Regulation of the genes encoding chloroplast antioxidant enzymes

The plastid antioxidant system (PAS) is a highly cooperative network of peroxidases, superoxide dismutases, low molecular weight antioxidant and their regenerating enzymes [84, 85], in which even the loss of the strongest expressed components or the components with the highest peroxidase activity can be compensated by support of remaining ones over time [86, 87]. Previous analysis demonstrated that the composition of the PAS is temperature-regulated [18, 23, 88]. In 3 week old Col-0 plants, *CSD2* (*CuZn-SUPEROXIDE DISMUTASE 2*), *2CPB* (*2-CYS PEROXIREDOXIN B*) and *GPX1* (*GLUTATHIONE PEROXIDASE 1*) transcript levels increase gradually with temperature [88]. Due to independent cold regulation, *sAPX* (*STROMAL ASCORBATE PEROXIDASE*) and *GPX7* (*GLUTATHIONE PEROXIDASE 7*) transcript levels increase in response to shifts from 20 to 10 °C as well as from 20 to 30 °C. *tAPX* (*THYLAKOID-BOUND ASCORBATE PEROXIDASE*) transcript levels decrease upon both temperature shifts [88]. In 6-week old plants, also *2CPA* (*2-CYS PEROXIREDOXIN A*) transcript levels increased at 4 °C [23]. Comparison of 10 Arabidopsis accessions revealed that the individual PAS composition achieved by cold acclimation affects chloroplast ROS levels in the post-cold response [23]. Most PAS enzymes are prone to oxidative inactivation [89–91] and need to be steadily replaced by de-novo synthesis. Due to the high expression intensity, even slight relative changes in transcript abundance of PAS genes result in strong absolute effects.

**Fig. 6:**
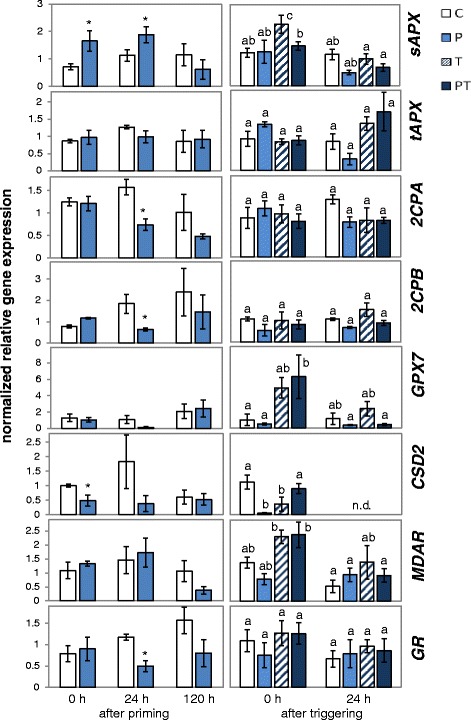
The transcript levels of genes for chloroplast antioxidant enzymes immediately, 24 h and 120 h after priming and immediately and 24 h after 24 h triggering at 4 °C in LTC-primed plants. The transcript levels were normalized to the transcript levels of naïve plants before the on-set of priming. Significant differences between primed and unprimed plants (Student’s t-test; *p* < 0.05, *n* = 3–6) are labelled with an asterisk in the left part of the figure. After triggering, significantly different data (Tukey’s test; *p* < 0.05, *n* = 3–6) are labelled with different letters. The samples were labelled as in Fig. 1

**Fig. 7:**
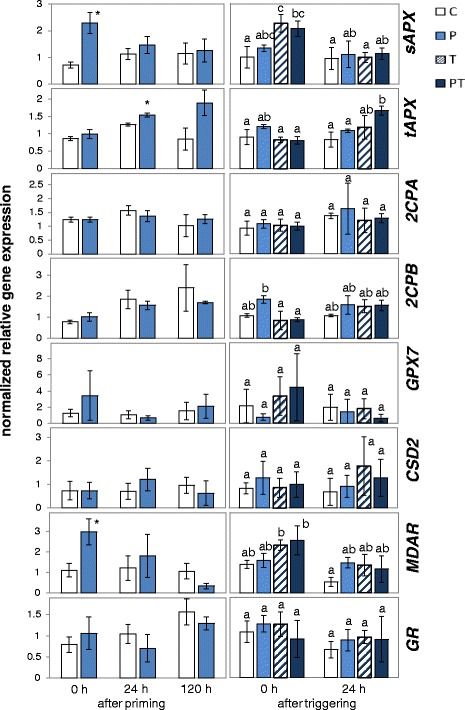
The transcript levels of genes for chloroplast antioxidant enzymes immediately, 24 h and 120 h after priming and immediately and 24 h after 24 h triggering at 4 °C in STC-primed plants. The transcript levels were normalized to the transcript levels of naïve plants immediately before the on-set of priming. Significant differences between primed and unprimed plants (Student’s t-test; *p* < 0.05, *n* = 3–6) are labelled with an asterisk in the left part of the figure. After triggering, significantly different data (Tukey’s test; *p* < 0.05, *n* = 3–6) are labelled with different letters

For further analysis, we compared regulation of the genes for the five most prominent chloroplast peroxidases, namely *sAPX*, *tAPX*, *2CPA*, *2CPB* and *GPX7*, of the gene for the main CuZn-superoxide dismutase, *CSD2*, and the transcript levels of the key genes for regeneration of the low molecular weight antioxidants ascorbate and glutathione in *Arabidopsis thaliana* var. Col-0, namely monodehydroascorbate reductase (*MDAR*) and glutathione reductase (*GR*) in response to 24 h (STC) and 2 week long chilling stress (LTC), during a 5 day long lag-phase at 20 °C and after triggering the plants for 24 h at 4 °C (Figs. 6 and 7). Gene expression regulation differed after STC- and LTC-priming:

#### Response to the first cold stimulus

Immediately after LTC-priming (Fig. 6; 0 days), *sAPX and 2CPB* transcript levels were increased and the mRNA levels of *CSD2* decreased. The effects were similar or stronger than the effects described previously for plants which were transferred from optimal growth temperatures to 10 °C [88]. After STC-priming, *sAPX* and *MDAR* transcript levels were significantly (*p* < 0.05) higher than prior to the cold treatment (Fig. 7; 0 days). The transcript levels of the other genes did not differ significantly.

#### Regulation in the lag-phase

After the first cold stimulus, the plants were re-transferred to optimal growth temperatures. In short-term cold (STC) treated plants (Fig. 7), *sAPX* transcript levels decreased within the first 24 h. Five days after the first treatment, the transcript level was indistinguishable from that in un-treated plants of the same age (C). *MDAR* levels decreased in response to STC (P-plants) below the control levels. *tAPX* levels increased to twice the levels of C-plants. *2CPA*, *GPX7* and *CSD2* levels did not change significantly. *GR* and *2CPB* increased in C-plants due to developmental regulation during the lag-phase. In P-plants the increase was less.

After long-term cold pretreatment (LTC) (Fig. 6), *sAPX* and *MDAR* transcript levels decreased in P-plants from higher starting levels to lower ones than in the control plants (C). Despite a similar effect of 24 h and 2 week long chilling stress on *tAPX* directly after the cold-stress, the *tAPX* transcript levels did not increase in LTC-primed plants like it did in the STC-treated ones. *2CPA* and *GPX7* transcript levels, which were not regulated by STC, decreased (transiently) in LTC-treated plants.

In summary, both treatments resulted in a shift in the relative expression intensity of sAPx and tAPx: STC mainly increased *tAPX* expression, while LTC resulted in an increase in *sAPX* mRNA. In parallel, the transcript levels for *MDAR*, *GR* and *2CPA* decreased in LTC plants during the 5 days at 20 °C demonstrating that the duration of the cold stress strongly impacted on regulation of PAS genes for various days.

#### Response to the triggering cold stimulus

After 5 days at 20 °C, a 24 h cold pulse (triggering pulse) was applied to short- and long-term primed and unprimed plants (Fig. 1a). Comparison of the transcript levels between plants, which had only perceived the later cold stimulus (T-plants), with the levels in P-plants after STC-priming (Fig. 7) demonstrated that the general cold regulation was maintained for all genes during the experiment. Cold regulation overwrote most differences observed at the end of the lag-phase (Figs. 6 and 7; 120 h after priming and 0 h after triggering). Even strong effects, like the elevated *tAPX* mRNA levels or the decreased *MDAR* transcript levels in 24 h cold-treated P-plants or the decreased *MDAR*, *2CPA* and *GR* levels in 2 week long cold-treated P-plants, were compensated in response to the triggering stimulus (Figs. 6 and 7).

The comparison of primed and triggered (PT) and only triggered (T) plants did not show priming effects on PAS genes in the STC-primed plants (Fig. 7). In the LTC-primed plants, *sAPX* transcript levels were significantly decreased in PT-plants relative to T-plants immediately after the end of the triggering stimulus (Fig. 6).

### Regulation of sAPX and tAPX protein level

**Fig. 8:**
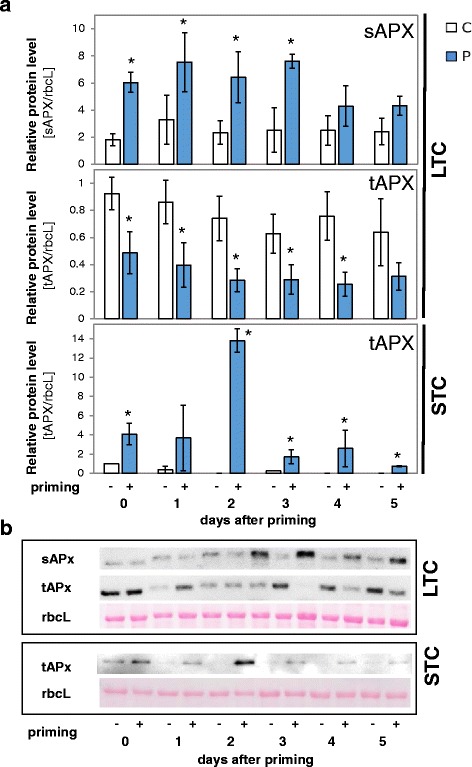
sAPX and tAPX protein levels in the lag-phase after LTC and STC. The proteins were detected with an antibody against the stromal part of Arabidopsis tAPX, which recognizes chloroplast and most cytosolic APXs. **a** The band intensities were quantified on 3–4 blots after luminometric detection. The data were analyzed by ANOVA (Student t-test; *p* < 0.05, *n* = 3–4). Significant differences between primed and unprimed plants are labeled with an asterisk. Data on primed plants are shown in blue, those obtained with naïve plants in white. **b** Examples of luminograms obtained by Western-Blotting with the APx antibody in STC- and LTC-treated plants and a digital photo of the PonceauS stained rbcL on the same membranes prior to the incubation in the antibody solution

To test whether the STC- and LTC-induced shifts in the *sAPX* and *tAPX* transcript levels (Fig. 6 and 7) influence the sAPX and tAPX protein levels, proteins were isolated at various time-points during the lag-phase and analyzed with antibodies against the mature, stromal domain of Arabidopsis tAPX on Western blots. The chloroplast APX isoforms were identified by comparison of the band patterns with the band patterns of *sAPX* and *tAPX*-knock-out lines [86]. For quantitative analysis, the samples were standardized on the protein levels of the large subunit of Ribulose-1,5-bisphosphate-carboxylase-oxygenase (RUBISCO) (*rbcL*) (Fig. 8).

The changes in transcript abundance (Figs. 6 and 7) were reflected in the protein patterns in both plant sets (Fig. 8): LTC increased the sAPX protein levels and deceased the tAPX ones. After STC, the tAPX protein levels were higher in cold-treated plants during the entire lag-phase. The differences were strongest 2 days after the first cold treatment.

### Response of ROS-marker genes in sAPX and tAPX-knock-out lines

**Fig. 9:**
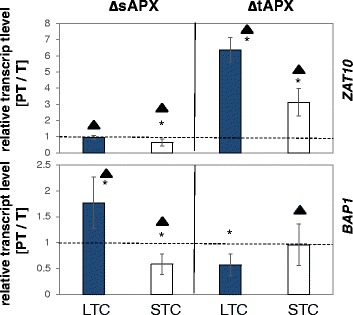
Relative *ZAT10* (*top*) and *BAP1* (*bottom*) transcript levels in ΔsAPX and ΔtAPX lines (knock-out lines) of *Arabidopsis thaliana* after LTC- (*dark blue bars*) or STC-priming (*white bars*) and triggering with a 24 h cold stimulus. The values in PT-plants were divided by the values of T-plants. The data were analyzed by ANOVA (Tukey’s test; *p* < 0.05, *n* = 3). Significant increases and decreases relative to “PT = T” (dotted line) are labeled with an asterisk. Significantly lower inhibition of transcript induction relative to the response in Col-0 (Fig. 4) (Student’s t-test; *p* < 0.05, *n* = 3–6) are labeled with a black triangle

To test the causality between *sAPX* and *tAPX* expression on the priming of *ZAT10* and *BAP1* regulation, we quantified *ZAT10* and *BAP1* transcript levels in *sAPX*- and *tAPX*-knock-out lines of *Arabidopsis thaliana* (ΔsAPX, ΔtAPX) after STC and LTC (Fig. 9). As previously analyzed [86], lack of *tAPX* promotes *sAPX* expression upon moderately increased light intensities (600 μmol photons m^−2^ s^−1^) and at 10 °C. Lack of sAPX only increases the *tAPX* expression at elevated light intensities (600 and 1300 μmol photons m^−2^ s^−1^), but not in the cold.

Our analysis revealed that the priming-dependent inhibition of *ZAT10* induction (as observed in Col-0; Fig. 4) was inverted to stronger induction by the triggering 4 °C stimulus in STC- and LTC-primed plants than in unprimed plants in the ΔtAPX-lines (Fig. 9). Priming-dependent regulation of *BAP1* (Fig. 4) was lost in STC-primed ΔtAPX-plants (Fig. 9) demonstrating a regulatory effect of tAPX on both ROS-marker genes. Lack of sAPX resulted also in a loss of the priming effect in LTC-plants, but only a weak effect in STC-plants (Fig. 9). *BAP1* showed stronger responses in LTC pretreated plants and a less severe negative effect in STC-primed plants (Fig. 9) than in Col-0 (Fig. 4).

### ROS levels

**Fig. 10:**
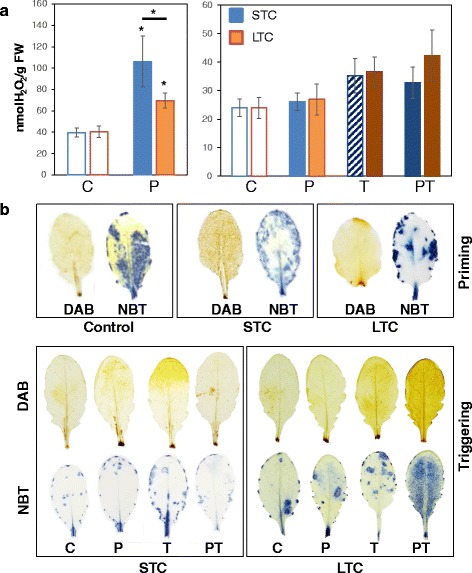
**a** H_2_O_2_ levels in rosette leaves after STC (*light* / *dark blue*) and LTC-priming (*orange / brown*), in the lag-phase and 24 h 4 °C triggering. The data were analyzed by ANOVA (Tukey’s test; *p* < 0.05, *n* = 10). Asterisks mark significantly different data. **b** DAB and NBT staining patterns of fully light exposed upper, fully developed rosette leaves in control plants (C), only primed (P), only triggered (T) and primed and triggered (PT) plants after the cold treatment

Since the gene expression data pointed out a regulatory function of chloroplast APX regulation, we analyzed the H_2_O_2_ levels in the STC- and LTC-plant sets. Directly after the first cold treatments, the H_2_O_2_ levels were increased (Fig. 10a). The effect was stronger after STC (2.7-fold) than after LTC (1.7-fold), which enables acclimation. In the control plants, the H_2_O_2_ levels decreased between the time-point the other plants were primed and triggered due to developmental regulation, such as a decrease in H_2_O_2_-mediated cell wall biosynthetic activity after full leaf expansion. The 24 h cold stimulus used for triggering increased them 1.5-fold (T-plants). LTC-primed plants (PT-plants) accumulated by average slightly more H_2_O_2_ in response to triggering than the unprimed T-plants, and STC-primed slightly less (PT-plants), although the effects were not significant due to strong biological variation (Student’s t-Test *p* < 0.1; *n* = 15 – 20).

Additionally, rosette leaves were stained with 3, 3′-diaminobenzidine (DAB) for H_2_O_2_ and nitroblue tetrazolium (NBT) for free radicals (Fig. 10b). ROS-dependent DAB polymerization amplifies the small differences observed in the linear colorimetric assay used for H_2_O_2_ quantification (Fig. 10a). DAB-staining confirmed accumulation of H_2_O_2_ all over the leaf blades after priming (Fig. 10b) and demonstrated higher H_2_O_2_-levels after STC than after LTC priming. The second cold-treatment increased the H_2_O_2_ levels stronger in LTC-primed plants and less in STC-primed plants. NBT-staining demonstrated accumulation of free radicals only for LTC-treated PT-plants (Fig. 10b).

### Analysis of priming specificity

**Fig. 11:**
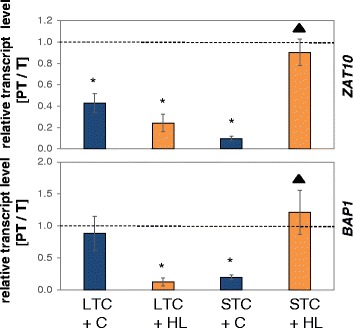
Relative *ZAT10* (*top*) and *BAP1* (*bottom*) transcript levels after LTC- or STC-priming and triggering with a 24 h cold stimulus (“+ C”: *dark blue*) or a high-light stimulus (“+ HL”: *orange*). The values in PT-plants were divided by the values of T-plants. The data were analyzed by ANOVA (Tukey’s test; *p* < 0.05, *n* = 3). Significant increases and decreases relative to “PT = T” (*dotted line*) are labeled with an asterisk. Significantly lower inhibition of transcript induction relative to the response in Col-0 (Figs. 3 and 4) are labeled with a black triangle

*ZAT10* and *BAP1* can be alternatively induced by high light and cold [52, 56, 92]. To test whether the cold-priming effect on chloroplast-to-nucleus ROS signaling is cold-specific or also light-regulated, we compared the response of LTC- and STC-primed plants after triggering them either 24 h at 4 °C at control light intensity or 24 h at control temperature, but 2.5-fold higher light intensity (Fig. 11). After STC-priming, the triggering cold stimulus blocked *ZAT10* and *BAP1* induction (Fig. 11), as shown before (Figs. 6 and 7). After triggering with the higher light intensity, the genes were induced to the same extent in PT- and T-plants showing that STC-priming did not affect the response to high-light triggering.

In LTC-primed plants, higher light intensity and cold blocked *ZAT10* induction similarly and elevated light blocked *BAP1* even stronger (Fig. 11). Consequently, the triggering response on *ZAT10* and *BAP1* was not as specific to cold after LTC-priming as it was after STC-priming.

## Discussion

**Fig. 12:**
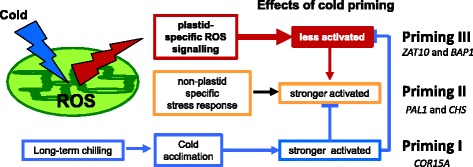
Model depicting our hypothesis on cold-priming in Arabidopsis. Cold priming results in less activation of chloroplast-to-nucleus ROS signaling by a short triggering cold stimulus (priming III) in parallel to stronger activation of non-plastid specific stress response genes (priming II) and cold responses (priming I). Upon prolonged cold periods, cold acclimation is induced and weakens the priming effects

Many organisms have adapted the competence to memorize experiences. While “learning” depends on acquiring knowledge by experience or cognitive activity, “priming” is a (widely implicit) process by which earlier contact with a stimulus manifests a “memory” and affects the conversion of future stimuli into responses [93]. Response priming was originally defined in psychology [94] and was later shown to prepare also plants for future stress [25, 27]. Our study showed positive priming of *COR15A*, *PAL1*, *CHS* and negative priming of *BAP1* and *ZAT10* (Fig. 4). The priming effects on *BAP1* and *ZAT10* were more pronounced after STC than LTC (Fig. 4). *PAL1* was less and *CHS* similarly induced after STC as compared to LTC, while stronger induction of *COR15A* was specific for LTC-priming. The different responses suggest three parallel acting cold-induced priming processes (Fig. 12):

### Priming I: The priming effect on COR15A requires long-term cold

*COR15A* can be induced by drought, alternatively to cold [71, 95]. *RD29B* (*RESPONSIVE TO DESICCATION 29B*) and *RAB18 (RESPONSIVE TO ABA 18)* memorize previous drought stress by H3K4me3 histone demethylation [96]. In the same experiment, *COR15A* was not primable and has been designated to fall in the class of “non-memory genes” [96]. Later, Kwon et al. [60] demonstrated that cold-induced H3K27me3 histone demethylation does not lead to stronger *COR15A* activation by triggering 2 day cold treated Arabidopsis for 24 h with a 4 °C cold stimulus after a 1 day long lag-phase. In our study, *COR15A* was also not more cold-induced in STC-primed plants than in unprimed plants by a 24 h cold stimulus 5 days after the first one (Fig. 4). However, *COR15A* was stronger expressed after LTC-priming (Fig. 4). Φ_PSII_, which is a sensitive indicator for photoinhibition and imbalances from photostasis [97, 98], was not changed immediately after triggering (Fig. 3) excluding *COR15A* induction by persisting cold acclimation responses. Photochemical quenching (qP) was stronger activated in LTC-primed plants compared to non-primed and STC-primed plants 2–3 days after triggering (Fig. 3). The reaction indicated soft reactivation of cold acclimation responses after triggering [7, 11–14], such as changes in the thylakoid membrane composition or chloroplast carbon metabolism [8, 22, 99]. This conclusion is supported by the observation that *BAP1* expression was also slightly increased and very “noisy” after the LTC treatment. The gene is regulated by ICE1 (INDUCER OF CBF EXPRESSION 1), which is a key regulator of the ICE1-CBF-COR regulon [100].

A comparison of post-cold regulation in a series of Arabidopsis accessions linked low PAS activity after 14 days at 4 °C with higher post-cold *CBF* expression and soft prolonged activity of cold acclimation responses [23]. In LTC-primed plants, the tAPx protein levels were decreased for days after the cold treatment (Fig. 8). In response to triggering, the plants accumulate more ROS (Fig. 10), which can explain higher *COR15A* expression as a response to stronger ROS-dependent activation of the primary ICE-CBF-COR cold response regulon [63].

### Priming II: Stronger activation of CHS and PAL1

*PAL1 and CHS* were stronger cold-activated after LTC-priming, and to a lesser extent also after STC-priming, than in not cold-pretreated plants. *PAL1* is stronger inducible after priming Arabidopsis leaves with the disease resistance activator benzothiadiazole [58]. The “stress memory” was shown to depend on the MAP-kinases MPK3 and MPK6 [58], which are key elements of receptor-mediated signaling [101] and respond to ROS signals [102, 103]. As a major integration point of plant stress signaling [104], MPK6 is also involved in cold-regulation of gene expression [105] and the control of *CHS* transcription [59]. Activation of *CHS* and *PAL1* supports (iso-)flavonoid and anthocyanin biosynthesis [106], cell wall stabilization and wound-healing, shields photoreaction centers from light and promotes antioxidant protection [39, 107, 108]. The abiotic stress signaling pathway and pathogen signaling share, beside MPK6, e.g., cytosolic calcium signaling, activation of plasma membrane redox activities and signal initiation by cellular redox imbalances / ROS [109–111]. The common signal transduction pathways of cold and pathogen signaling and priming response of *CHS* and *PAL1* after cold (Fig. 4) and pathogen-attack simulating priming [58] indicate a pleitropically inducible priming pathway.

### Priming III: Suppression of BAP1 and ZAT10 activation

In contrast to the concept of having stronger activation of stress-response pathways in primed plants [29], *BAP1* and *ZAT10* were less expressed after cold-priming (Fig. 4). The effect was more pronounced after STC than LTC priming (Fig. 4). Primary cold acclimation responses or reactivation of cold responses upon triggering might have suppressed cold-induction of the genes. The assumption is contradicted by the observation that *ZAT10* is stronger expressed after two 24 h 4 °C pulse separated by a lag-phase of only 3 days [22]. In the experiment by Byun et al. [22], the transcriptome showed a stronger cold-imprint than unprimed controls in response to a 24 h cold stimulus after the short lag-phase. Transcripts of genes involved in cold-acclimation of photosynthesis, chlorophyll metabolism and synthesis of ascorbate accumulated during triggering. After a 5-day long lag-phase, we observed in our STC-experiment no effect on *COR15A* cold-induction (Fig. 4). Our data also do not indicate differences in pigmentation, photosynthetic-effects (Figs. 2 and 3) or buffering effects of ascorbate (Fig. 5). We conclude that *ZAT10* and *BAP1* cold induction were (partly) blocked 5 days after STC-priming due to priming-specific regulation and that activation of cold acclimation antagonizes the priming effect.

Specific metabolites or ROS-modified proteins might store the priming information [112, 113]. However, mechanisms such as e.g., thiol oxidation or metabolite accumulation cannot explain why STC results in stronger priming effects than LTC (Fig. 4). F_V_/F_M_ and photochemical and non-photochemical quenching data (Fig. 3) also exclude ROS damage of the photoreaction centers for both priming treatments. Signaling is widely controlled by lower ROS doses than those causing damage and affecting F_V_/F_M_ [8]. In chloroplasts, ROS are mainly generated at the thylakoid membrane, where tAPX provides antioxidant protection [114, 115]. Accumulation of ascorbate and sAPX in the stroma (Figs. 5, 7 and 8) of LTC-primed plants was not sufficient to control ROS levels (Fig. 10), but accumulation of tAPx after STC-priming. The data indicate that priming ROS-signaling is locally controlled at the thylakoid membrane by tAPx. If ROS can escape antioxidant protection at the thylakoids [116, 117], they diffuse into the stroma. Second messengers can be formed due to the high reactivity of ROS. In addition to escaping ROS, they can subsequently activate a wider range of signaling cascades [118–120]. Consistently, here, we observed stronger induction of non-chloroplast specific ROS-regulated genes like *CHS* and *PAL1* (and *COR15A*) in LTC-primed plants as compared to STC-primed ones (Fig. 4). We conclude that the suborganellar distribution of ascorbate peroxidases controls priming regulation and is more important for the control of plastid ROS signaling than the total plastid antioxidant activity.

The analysis of gene regulation in ΔsAPX and ΔtAPX lines of Arabidopsis supported this hypothesis. After STC-priming, the priming effect on the triggering response of *ZAT10* was slightly decreased in ΔsAPX lines, but inverted in ΔtAPX lines. The LTC-priming experiment showed no relevance of sAPX and showed strong tAPX dependency, despite down-regulation of *tAPX* expression in the lag-phase (Fig. 8), underlining the importance of tAPX for priming regulation. Non-induction of the ROS-regulated genes *ZAT10* and *BAP1* (Fig. 4) correlates with lower H_2_O_2_-levels and higher *tAPx* expression after STC-priming. tAPX abundance is regulated during the lag-phase (Figs. 7 and 8). We conclude that tAPX-mediated thylakoid protection serves as a priming hub, which stores information on priming over time.

### What is the benefit of lower induction of ROS-response regulators after STC-priming?

Activation of genes for extra-plastidic antioxidant enzymes by chloroplast-derived ROS was one of the first indications for chloroplast-to-nucleus ROS-signaling [121]. This process, or at least a significant part of it, is mediated by ZAT10 [56, 79]. It is tempting to question how plants can benefit from lower induction of *ZAT10* after priming. In their detailed characterization of ZAT10 function, Rossel et al. [56] postulated coexistence of high-light controlled systemic acquired acclimation (SAA) and pathogen stimulated systemic acquired resistance (SAR). These systemic effects, like cold-priming, prepare / prime plants for future stress [28]. Mittler et al. [79] showed in *ZAT10* gain- and loss-of-function mutants that decreased expression of the transcription factor enhances stress tolerance of Arabidopsis. Lower expression of *BAP1* further supports activation of immunity responses [122, 123]. We postulate that the priming-dependent suppression of stress signal mediator genes, such as *ZAT10* and *BAP1*, is a strategy to promote induction of pleiotropic stress protection. Here, *PAL1* and *CHS*, which support biosynthesis of versatile secondary stress protection mediators, such as salicylate, lignin and flavonoid- and anthocyanidin [107, 124], were stronger induced after priming (Fig. 4).

This study demonstrated that PAS regulation is independent from activation of cold acclimation (Figs. 6 and 7). PAS-mediated priming of chloroplast ROS signaling (priming III) might be of special importance in spring when the average temperatures increases and is most of the time above those necessary for *ICE1*-*CBF3*-*COR*-regulon-mediated cold-protection, but plants are challenged by unpredictable short cold periods.

## Conclusions

Priming Arabidopsis at cold temperatures resulted in decreased activation of marker genes for chloroplast-to-nucleus ROS signaling and in stronger activation of *COR15A*, *PAL1* and *CHS*. Our data pointed out *tAPX* as priming mediator. We conclude that PAS-controlled cold-priming counteracts activation of genes mediating chloroplast-to-nucleus signal transduction to support stress responses independent of acclimation.

## Methods

### Plant materials and growths conditions

*Arabidopsis thaliana* var. Col-0 plants and *sAPX* and *tAPX*-knock out lines [86] were grown individually in pots (6 cm diameter) on Arabidopsis soil [70 volumes “Topferde” (Einheitserde, Sinntal-Altengronau, Germany), 70 volumes “Pikiererde” (Einheitserde, Sinntal-Altengronau, Germany), 25 volumes Perligran Classic (Knauf, Germany)] supplemented with 0.5 g l^−1^ dolomite lime (Deutsche Raiffeisen-Warenzentrale, Germany) and 0,5 g l^−1^ Axoris Insekten-frei (COMPO, Münster, Germany) in a growth chamber at a day / night temperature of 20 ± 2 °C and 18 ± 2 °C, respectively and a 10 h light / 14 h dark cycle. Illumination with 100–110 μmol photons*m^−2^*s^−1^ was performed with L36W/840 Lumilux Cool White fluorescent stripes (Osram, Munich Germany). The relative humidity was 60 % ± 5 % during day and night.

For stratification, the seeds were kept for three days at 4 °C in darkness on wet Arabidopsis soil before they were transferred to the growth chamber. At an age of 7–8 days, seedlings were transferred individually to 6 cm-pots. For cold treatment, the plants were relocated for 24 h or 2 weeks to 4 °C 2.5 h after onset of light for priming and cultivated in the same 10 h light / 14 h dark pattern in a growth room with the same illumination and aeration setting and humidity control. The light intensity (which is lower with the same fluorescence stripes at lower temperature) was adjusted by placing the plants closer to the light source and controlled by measuring the light intensities at least once a week. The leaf top temperature was controlled with an infrared thermometer. For triggering, the primed plants and control plants were transferred 5 days after priming for 24 h to 4 °C 2.5 h after onset of light. Afterwards the plants were cultivated at a day / night temperature of 20 ± 2 °C and 18 ± 2 °C until harvest. For high-light treatments, the light intensity was increased 2.5-fold. The standard growth room and shelf aeration control system was sufficient to maintain the leaf-top temperature (as controlled with an infrared thermometer).

### Determination of fresh weight and leaf numbers

Whole rosettes were harvested seven days after triggering. The fresh weight was determined immediately by weighing the plants. The leaf numbers were counted afterwards.

### Determination of chlorophyll levels

Chlorophyll contents were quantified from fresh plant material according to Porra [125] in 80 % acetone supplemented with a trace of CaCO_3_. The extraction was carried out over night at −20 °C. All samples were taken 2.5 h after onset of light.

### Determination of H_2_O_2_ levels

H_2_O_2_ levels were quantified after extraction of 50 mg plant material in 200 μl 5 mM KCN and 15 min centrifugation at 13.000 g at 4 °C according to Gay [126]. 100 μl of the supernatant were mixed with 1 ml of the dye solution (100 volumes 125 μM xylenol orange in 100 mM sorbitol freshly mixed with 1 volume of 25 mM (NH_4_)_2_Fe(SO_4_)_2_ in 2.5 M H_2_SO_4_). The H_2_O_2_ concentration was calculated from the absorbance at 560 nm after 15 min at room temperature based on a H_2_O_2_ standard curve.

### DAB and NBT staining

Freshly harvested leaves were incubated in NBT (1 mg / ml nitroblue tetrazolium in 10 mM NaN_3_, 8 % (w/v) NaCl, 0.2 % (w/v) KCl, 1.44 %(w/v) Na_2_HPO_4_ and 0.24 % (w/v) KH_2_PO_4_; pH 7.4) or 3,3- DAB buffer (1 mg/ml diaminobenzidine in 8 % (w/v) NaCl, 0.2 % (w/v) KCl, 1.44 % (w/v) Na_2_HPO_4_ and 0.24 % (w/v) KH_2_PO_4_; pH 7.4). After staining in darkness, the background was removed in a 1:1:3 mixture of acetic acid, glycerol and ethanol at 60–80 °C.

### Chlorophyll-a fluorescence analysis

The maximum quantum efficiency of PS-II (F_V_ / F_M_), the effective quantum yield (ϕ_PSII_), photochemical quenching (qP) and non-photochemical quenching (NPQ) were determined by chlorophyll-a fluorescence analysis using a MINI-PAM fluorimeter (Walz, Effeltrich, Germany). For determination of the maximum quantum yield (F_V_ / F_M_ = (F_M_ – F_0_)/F_M_; [45]) the plants were dark acclimated for 20 min. The measurement was performed with a saturating light flash (1300 μmol photons m^−2^ s^−1^). Photochemical quenching (qP = (F_M´_ - F)/(F_M´ -_ F_0´_); [48]), non-photochemical quenching (NPQ = (F_M_ /F_M´_) – 1; [49]) and the effective quantum yield of photosystem II (ϕ_PSII_ = (F_M´_- F)/F_M´_; [46]) were determined with 8 saturating light flashes spaced 30 s and an actinic light intensity of 3-times the growth light intensity.

### Western blot analysis

Protein levels were determined with BIO-RAD DC Protein Assay (Bio-Rad, Munich, Germany) according to manufacturer’s instructions. Gel electrophoresis and Western blotting were performed with 12 % acrylamide gels as described in [88]. The transfer efficiency was checked by staining of the membranes with 0.2 % (w/v) Ponceau S in 3 % (v/v) acetic acid. For detection a serum was generated by immunization of a rabbit with heterologous expressed thylakoid-bound ascorbate peroxidase lacking the chloroplast import signal and the transmembrane helix. The antibody binding was visualized luminometrically in a ImageQuant LAS4000-mini (GE HealthCare, Uppsala Schweden) with peroxidase anti-rabbit IgG conjugate (Sigma Aldrich, Taufkirchen, Germany) and Pierce ECL Western Blotting Substrate (Thermo Scientific, Dreieich, Germany).

### Quantitative real-time PCR

Entire rosettes of 4–5 plants (per sample) from 3 to 5 independently grown STC- and LTC-plant sets (C-, P-, T- and PT-plants) were collected prior to, immediately after and 1 and 5 days after the cold-treatments, immediately frozen in liquid nitrogen and stored at −80 °C. Total RNA was extracted from 100 mg of ground plant material using the GeneMatrix Universal RNA Purification Kit (EURx, Gdansk, Poland) according to the manufacturer’s instruction (including the optional DNase digestion with 1 U/μl DNase I (Fermentas, St. Leon-Rot, Germany)). Only RNAs with an A_260nm_/A_280nm_ between 1.8 and 2.0 were used for further analysis. The RNA integrity was assessed by RNA gel electrophoresis using a 1 % agarose gel supplemented with 0.9 % formaldehyde.

cDNA was synthesized using the High Capacity Reverse Transcription Kit (Applied Biosystems, Carlsbad, CA) and 10 μM oligodT_16_V primer. Each reaction (20 μl) contained 2 μg of RNA and 20 U of reverse transcriptase and was incubated for 10 min at 25 °C, for 2 h at 37 °C and for 5 min at 85 °C. The samples were tested for DNA contamination with primers flanking two short introns and an exon of the 2CPA gene (At3g11630) (ATbas-O1H GACTTTACTTTCGTCTGC; ATbas-O4H ATCACTCCTTCCTTGTCG) by DNA gel electrophoresis after 40 cycles.

Real time quantitative polymerase chain reaction (qRT-PCR) was performed in 20 μl containing 50 ng template cDNA, 2 μl 10× buffer (160 mM ammonium sulfate, 1 M Tris–HCl pH 8.3, 0.1 % (v/v) Tween-20, 0.8 μl 50 mM MgCl_2_, 0.4 μl 5 mM dNTP, 0.2 μl 10× SYBR Green (Sigma-Aldrich, Germany), 0.04 μl 5 U/μl OptiTaq Polymerase (EURx, Gdansk, Poland) and 0.12 μl of 50 μM gene-specific primers (300 nM final concentration). The primers and the accession numbers of the analyzed genes are listed in Table 1. All reactions were performed in triplicates per biological sample on a CFX96 real-time System (Bio-Rad, Hercules, CA). The cycling conditions were: 5 min at 95 °C, followed by 40 cycles 95 °C/ 15 s, 60 °C/ 30 s and 72 °C/ 30 s. All primers, if applicable, were designed to span exon–intron border using the QUANTPRIME software [127] to prevent the amplification of genomic DNA. Primer specificity was assessed by inspection of the melting curves after 40 cycles. The C_t_ values were determined using the regression model within the CFX Manager software 3.0 and further analyzed for primer amplification efficiency. Normalization was done on the geometric mean of the *ACT2* (*ACTIN 2*) and the *YLS8* (*YELLOW LEAF SPECIFIC PROTEIN 8*) transcript levels [128].

**Table 1:** Primers used for qRT-PCR analysis

*Annotation*	*AGI code*	Forward	Reverse
*2CPA*	At3g11630	CCCAACAGAGATTACTGCCT	ATAGTTCAGATCACCAAGCCC
*2CPB*	At5g06290	TCATACCCTCTTCCTCGGCATC	ACCGACCAGTGGTAAATCATCAGC
*ACT2*	At3g18780	AATCACAGCACTTGCACCAAGC	CCTTGGAGATCCACATCTGCTG
*BAP1*	At3g61190	ATCGGATCCCACCAGAGATTACGG	AATCTCGGCCTCCACAAACCAG
*CHS*	At5g13930	TTCCGCATCACCAACAGTGAAC	CGCACATGCGCTTGAACTTCTC
*COR15A*	At2g42540	AACGAGGCCACAAAGAAAGC	CAGCTTCTTTACCCAATGTATCTGC
*CSD2*	At2g28190	CTCAACAGGACCATTTCAACC	ATTGTTGTTTCTGCCAACGCCA
*GPX7*	At4g31870	CGTTAACGTTGCGTCAAGATGTGG	TGACCTCCAAATTGATTGCAAGGG
*GR*	At3g54660	GAAATTCCGCAAAGACTCCTC	CAGACACAATGTTCTCCTTATCAG
*MDAR*	At1g63940	TGGGAGAAACAGTGGAGGTTGG	TGGTAGAAGCTGGAACTCCTCAG
*PAL1*	At2g37040	GCAGTGCTACCGAAAGAAGTGG	TGTTCGGGATAGCCGATGTTCC
*sAPX*	At4g08390	AGAATGGGATTAGATGACAAGGAC	TCCTTCTTTCGTGTACTTCGT
*tAPX*	At1g77490	GCTAGTGCCACAGCAATAGAGGAG	TGATCAGCTGGTGAAGGAGGTC
*YLS8*	At5g08290	TTACTGTTTCGGTTGTTCTCCATTT	CACTGAATCATGTTCGAAGCAAGT
*ZAT10*	At1g27730	TCACAAGGCAAGCCACCGTAAG	TTGTCGCCGACGAGGTTGAATG

### Determination of ascorbate concentrations and the redox state

The ascorbate content and the redox state of the ascorbate pool (reduced ascorbate / total ascorbate) were determined as described in Baier et al. [87].

### Statistical analyses

Statistic test were performed with SPSS V22 (ANOVA, Tukey test, *p* < 0.05) and Microsoft Excel (Student’s T-test; *p* < 0.05).

## Abbreviations

2-CP, 2-Cys peroxiredoxin; 2CPA, 2-Cys peroxiredoxin A gene; 2CPB, 2-Cys peroxiredoxin B gene; APX, ascorbate peroxidase; C, control plants; CBF, C-repeat binding factor; Col-0, *Arabidopsis thaliana var. Columbia-0*; CSD2, CuZn superoxide dismutase 2; DAB, diaminobenzidine; LHCP, light-harvesting complex protein; LTC, long-term chilling; MAP, mitogen activated protein; MAPKK, mitogen-activated protein kinase kinases; MPK, MAP protein kinase; NBT, nitroblue tetrazolium; P, primed plants; PAS, plastid antioxidant system; PET, photosynthetic electron transport; PS-II, photosystem II; PT, primed and triggered plants; qRT-PCR, real-time quantitative polymerase chain reaction; ROS, reactive oxygen species; RUBISCO, ribulose-1,5-bisphosphate carboxylase; sAPX, stromal ascorbate peroxidase; STC, short-term chilling; T, plants which only faced the triggering stimulus; tAPX, thylakoid-bound ascorbate peroxidase
